# Determination of the Insecticidal Activities of *Beauveria bassiana* (Ascomycota: Hypocreales) Secondary Metabolites on *Dendroctonus micans* (Coleoptera: Curculionidae), *Glyphodes pyloalis* (Lepidoptera: Pyralidae) and *Thaumetopoea pityocampa* (Lepidoptera: Thaumetopoeidae)

**DOI:** 10.1007/s11686-026-01318-w

**Published:** 2026-06-08

**Authors:** Ali Soydinç, Birgül Özcan

**Affiliations:** 1 Trabzon Regional Directorate of Forestry, Trabzon, Turkey; 2 Faculty of Sciences and Letters, Biology Department, Mustafa Kemal University, Antakya, Hatay Turkey

**Keywords:** *Beauveria bassiana*, Bioinsecticide, *Dendroctonus micans*, *Glyphodes pyloalis*, Secondary metabolites, *Thaumetopoea pityocampa*

## Abstract

**Background:**

Forests provide many ecological and economic functions, but they are increasingly exposed to the invasion of various harmful insects. The bark beetle *Dendroctonus micans* and pine processionary moth *Thaumetopoea pityocampa* are highly destructive insect pests that kill living trees, leading to significant reductions in forest productivity and widespread tree death. In addition to these, *Glyphodes pyloalis* is another tree pest that damages mulberries, which are economically important for silk yarn production.

**Method and Results:**

In this study, a strain (27b2) from *Beauveria bassiana* was isolated from the cambium pests *D. micans* and *Ips sexdentatus*, which were collected dead from their natural habitat. Then, it was identified by morphological and molecular tests. The isolates of *B. bassiana* secondary metabolites were extracted from its extracellular medium and mycelium with ethyl acetate and acetone and then the efficacy of crude extracts against insect pests (*D. micans* adults and larvae), (*Glyphodes pyloalis* larvae and *T. pityocampa* larvae) was evaluated at concentrations of 500, 2000, and 5000 ppm. Consequently, it was determined that the ethyl acetate and acetone extracts of *B. bassiana* 27b2 showed a 100% lethal effect on *D. micans* adults and larvae, while the acetone extract showed a 100% lethal effect on *T. pityocampa* larvae.

**Conclusion:**

The data suggest that the secondary metabolites of isolate 27b2 may be utilized as bio-pesticides for the control of these pests. Also, this is the first study examining the lethal effects of secondary metabolites extracted from *B. bassiana* on *T. pityocampa*, and *D. micans*.

## Introduction

Globally, pest-related damage affects considerably larger forest areas than forest fires, and biotic damage from pests can increase a forest’s vulnerability to abiotic damage [1]. The great European spruce bark beetle, *Dendroctonus micans* (Kugelann 1794) (Coleoptera: Curculionidae, Scolytinae) is a significant invasive pest threatening spruce forests from Britain to Siberia. It has caused notable outbreaks affecting Norway spruce *Picea abies* in Britain and France, and oriental spruce *Picea orientalis* in Georgia and Türkiye [2, 3]. Under the bark of trees, *D. micans* larvae damage the cambium, leading to the eventual desiccation of the trees [4]. In 1956, a *D. micans* infestation in Georgian spruce forests caused the death of tens of thousands of trees [5]. Subsequently, the insect entered Türkiye, resulting in the felling of approximately 7 million cubic meters of timber across 120,000 hectares and causing significant economic losses in the forestry sector [6]. The pine processionary moth, *Thaumetopoea pityocampa* (Denis and Schiffermüller, Lepidoptera: Thaumetopoeidae), is one of the most destructive insect pests in the Middle East, North Africa, and many Southern European countries, where its larvae cause defoliation in various *Pinus* and *Cedrus* species, occasionally leading to significant outbreaks [7, 8]. In several southern European countries, *T*. *pityocampa* exhibits periodic outbreaks characterized by an approximate six-year periodicity [9]. In Türkiye, over 1.5 million hectares could be affected by *T. pityocampa* larvae, and this pest has caused a reduction of 12–65% in the annual diameter increment of host trees [10]. Furthermore, the urticating hairs of the caterpillars can induce allergies, respiratory distress, and asthma in humans [11]. Sericulture plays very important roles in the economic income of many developing countries such as India and China. However, one of the biggest threats to sericulture is *Glyphodes pyloalis* [12, 13]. *G. pyloalis* has been observed to feed on the leaves of *Morus* sp*.* trees [14] as a major concern for silkworm breeders with its harmful effects on the host plant and the potential harm it causes to silkworms through its feces [15]. *G. pyloalis*, one of the biggest pests for mulberry, seriously threatens mulberry quality and quantity [16].

In previous periods, chemical-based methods were used in the fight against plant pests [17] while biological control methods are now the preferred approach. A range of living entities, including insects, birds, plants, bacteria, fungi, viruses and nematodes were used widely in these methods along with secondary metabolites [18].

Secondary metabolites are organic substances produced by living organisms for defensive purposes, as a result of the production of primary metabolites [19]. Secondary metabolites are crucial in the processes of infection and control. The synthesis of these substances by entomopathogenic microbes does not directly affect growth or reproduction; instead, they primarily facilitate adaptation to the environment. Entomopathogenic microorganisms secrete a diverse range of secondary metabolites that have the potential to be employed in biological control [20].

*B. bassiana* is capable of producing a range of secondary metabolites such as bassianin, bassianolide, bassiacridin, oosporein, and tenellin with antibiotic, antifungal and insecticidal properties [21]. These metabolites have been identified as immunomodulators with in vitro antimicrobial and antifungal activity [22]. The first molecule to be characterized for its natural insecticidal properties was beauvericin, which was initially extracted from the mycelium of *Beauveria bassiana* and subsequently from various other species, including *Fusarium* and *Paecilomyces* [23]. It was determined that oxalic acid, a secondary metabolite with acaricide activity, is produced by *Beauveria* sp*.*, *Lecanicillium* (*Verticillium) lecanii*, *Paecilomyces fumosoroseus*, and *Metarhizium anisopliae* species. This substance causes the dissolution of cuticular proteins and is also a virulence factor of phytopathogenic fungi [24]. Beauverolides are responsible for the destruction of the host organism, while destruxins primarily serve as antimicrobial agents [25]. Additionally, oosporein serves as an antibacterial agent; yet, it also induces infection, which can be ascribed to its capacity to reduce the quantity of insect hemocytes, hence modifying the humoral immune system [26]. Tenellin and bassianin are yellow pigments synthesized by *B. bassiana* and *B. tenella* [27]. They comprise a polyketide chain that is decreased by the amide of the tyrosine segment [28].

The present study was conducted in the context of biological control of the pest species specified above. Fungi were isolated from the cambium pests *D. micans* and *I. sexdentatus*, which were collected dead from forest areas in the Trabzon province. The entomopathogenic fungal isolate was identified based on morphological and molecular characteristics. Secondary metabolites of the fungus were extracted from both the extracellular medium and the mycelium. A significant advantage of utilizing crude extracts over isolated molecules is the presence of a multifaceted chemical matrix that facilitates synergistic interactions among secondary metabolites, thereby significantly enhancing the overall biological efficacy. By leveraging this inherent cooperativity, the extracts demonstrate superior biopesticidal potential and increased lethality against target forest pests compared to the application of individual bioactive constituents [29]. The insecticidal activity of the crude fungal metabolite extracts was evaluated by applying them to the larvae of *G. pyloalis* and *T. pityocampa* species, as well as to both adults and larvae of *D. micans* species.

## Materials and Methods

### Insects

The mulberry pest, *G. pyloalis*, used in the study was collected from mulberry trees on the Trabzon Regional Forestry Directorate campus. Adults and larvae of *D. micans* were collected from spruce trees on the Kayabaşı Plateau in the province of Trabzon and *T. pityocampa* was collected from specimens of *Pinus brutia*.

### Isolation and Culturation of Fungi

Fungi were isolated from the cambium-destroying insects, *D. micans* and *Ips sexdentatus*, collected dead from spruce forests affiliated with the Trabzon Forestry Regional Directorate. The surface disinfection of the insects was performed by immersing them in a 1% NaOCl solution for 2 min, followed by two consecutive rinses in sterile distilled water for 2 min each. Subsequently, the larvae were placed between sterile blotting papers and allowed to air-dry. Dead insects were placed in sterile petri dishes containing moistened sterile filter paper and incubated at 28 °C for 10 days to observe mycelial growth. The fungal isolates from dead insect samples were recovered using Sabouraud Dextrose Agar (SDA). Pure cultures were obtained through single-colony isolation followed by at least three successive subculturing steps on fresh SDA plates to ensure culture purity. Colony morphology was routinely monitored during incubation, and only morphologically uniform colonies were selected and maintained for further studies.

### Morphological Characterization and Molecular Identification of Fungi

The identification of fungal isolates was first monitored by MALDI-TOF MS (Matrix-Assisted Laser Desorption/Ionization Time-of-Flight Mass Spectrometry, Bruker Daltonics GmbH, Bremen, Germany) coupled with the Filamentous Fungi library 3.0 analysis (Bruker Daltonics). MALDI-TOF MS analysis was performed using mycelia (100 mg) harvested from monospore cultures of fungal isolates grown on specific culture media. The procedure involved total genomic DNA isolation followed by the application of the protocols strictly recommended by the manufacturer. Then, the selected fungal isolate was grown on slides using the Agar-Block Culture Method and then the slides were stained with lactophenol blue and examined under a light microscope [30]. To confirm the morphological characteristics and MALDI-TOF identification, a representative isolate of *B. bassiana* was molecularly characterized. The universal primers ITS5 (5′-GGAAGTAAAAGTCGTAACAAGG-3′) and ITS4 (5′-TCCTCCGCTTATTGATATGC-3′), as described by [31] were used for the amplification of the DNA region encoding ITS-1–5.8S–ITS-2 of the DNA sample, generating 580 bp products. PCR amplicons were sequenced by Macrogen (Netherlands) on the Illumina MiSeq platform and the ITS sequence of isolate (27b2) was deposited in GenBank. Phylogenetic analysis of the fungal ITS sequences was performed using Molecular Evolutionary Genetic Analysis software (MEGA 11) [32]. The phylogenetic relationship was inferred using the Neighbor-Joining Method [33] and with 1000 bootstraps run [34]. The evolutionary distances were computed using the Kimura 2-Parameter Method [35] and are in the units of the number of base substitutions per site.

### Secondary Metabolite Extraction

Synchronized culture of the fungal isolate was established by serial passaging on the Potato Dextrose Agar (PDA) plate at 28 °C for 10 days. Two pieces of 1 cm^2^ from the synchronized culture were introduced in Erlenmeyer flasks containing 150 ml of Potato Dextrose Broth (PDB) and shaken at 180 rpm at 28 °C for 10 days. At the end of the incubation period, metabolites were extracted from the cell-free culture supernatant using ethyl acetate and from the fungal biomass using acetone. Aliquots of the cell-free supernatant were mixed with ethyl acetate (5:3), agitated at 200 rpm for one hour, and then transferred to the separatory funnel. The culture filtrates were extracted twice with ethyl acetate and evaporated by a rotary evaporator under a vacuum at 40 °C. The crude extract prepared by dissolving the precipitate in Dimethyl Sulfoxide (DMSO) was stored at  − 20 °C [36, 37]. The wet mycelium was suspended in an equal volume of 70% acetone and agitated at 250 rpm for 24 h [36]. The organic phase was removed by rotary vacuum and then the remaining part was redissolved in 5% acetone for activity tests and stored at 4 °C. Acetone was selected as the solvent for mycelial extraction and as a carrier in bioassays due to its high efficiency in dissolving a wide range of both polar and non-polar fungal secondary metabolites. Furthermore, acetone is characterized by high volatility, which ensures rapid evaporation after application, thereby minimizing potential solvent-induced mortality or interference with the insect’s physiological response. To account for any minimal effects of the solvent, control groups were treated with an equal volume of the respective solvent (5% acetone or DMSO), and all mortality data were further corrected using Abbott’s formula.

### Bioassay

Three concentrations from each extract (500, 2000, and 5000 ppm) were used in bioassay experiments, and acetone or DMSO solutions were used for control groups. There were three replicates for each treatment and control. All treatments were carried out in 2-L plastic containers. The following methods were used to evaluate the efficiency of ethyl acetate and acetone extracts on *G. pyloalis*, *T. pityocampa*, and *D. micans* collected from their natural habitats.

#### Glyphodes pyloalis

Three ml of each concentration of extracts (500, 2000, and 5000 ppm) were sprayed on *Morus* sp*.* leaves, and then ten individuals of *G. pyloalis* larvae (4.-5. instar) were placed in each prepared plastic container and mortality rates were determined by checking every day for 10 days. *G. pyloalis* larvae were incubated under the conditions with 12:12 h of light/dark cycle, at 25 ℃, and 70% RH.

#### *Thaumetapoea pityocampa*

Pine leaves sprayed with 3 ml of both extract concentrations were used at this stage. Incubation of *T. pityocampa* larvae (3.-4. instar) was carried out under 12:12 h of light/dark cycle, 22 °C, and 60% RH conditions, and mortality of larvae was checked daily for 10 days.

#### *Dendroctonus micans*

*D. micans* larvae (3.-4. instar) and adults (96% female) were placed between spruce barks with a square hole (15 cm^2^) sprayed with 3 ml of extract each and then placed in a plastic container [38]. Ten larvae/adults of *D. micans* were treated by dipping in each concentration of fungal extracts for 20 s [36]. The number of dead larvae/adults was counted daily for 10 days. The treatments were carried out at 20 ℃, 60% RH, and a 12:12 light/dark cycle [39].

### Statistical Analysis

Statistical analysis was performed using GraphPad Prism 5 (ver. 5.0; Graph Pad Software Inc, USA) software. Mortality rates were corrected using the Abbott formula. Homogeneity of variance and data normality was determined by the Bartlett’s test and the Kolmogorov–Smirnov test, respectively. Non-parametric Kruskal–Wallis and Dunn multiple comparison tests were used for statistical comparisons. Probit regression analysis was performed with the “Finney’s Probit Analysis Spreadsheet Calculator” program to determine the metabolites concentration (LC_50_ and LC_90_) required to kill 50 and 90% of the insects [40, 41].

## Results

### Morphological and Molecular Identification

**Fig. 1:**
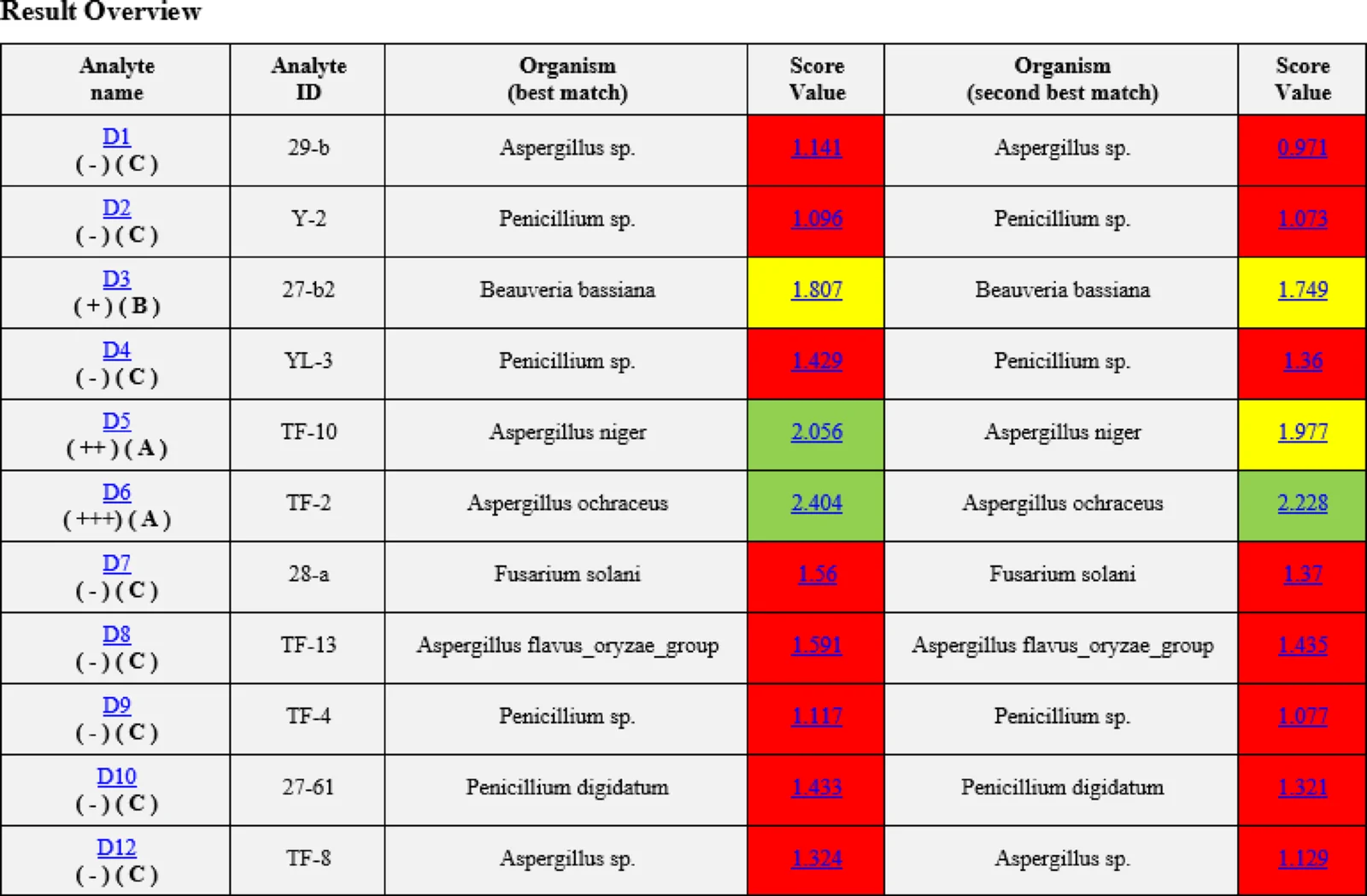
MALDI-TOF MS analysis score values for identification of fungal isolates

MALDI-TOF MS analysis was conducted on the 11 fungal isolates at the genus/species level. The analysis indicated that the isolate 27b2 matched the *Beauveria bassiana* in the MALDI-TOF library with a high similarity index (1.807). The study was therefore continued with this isolate (Fig. 1).

**Fig. 2:**
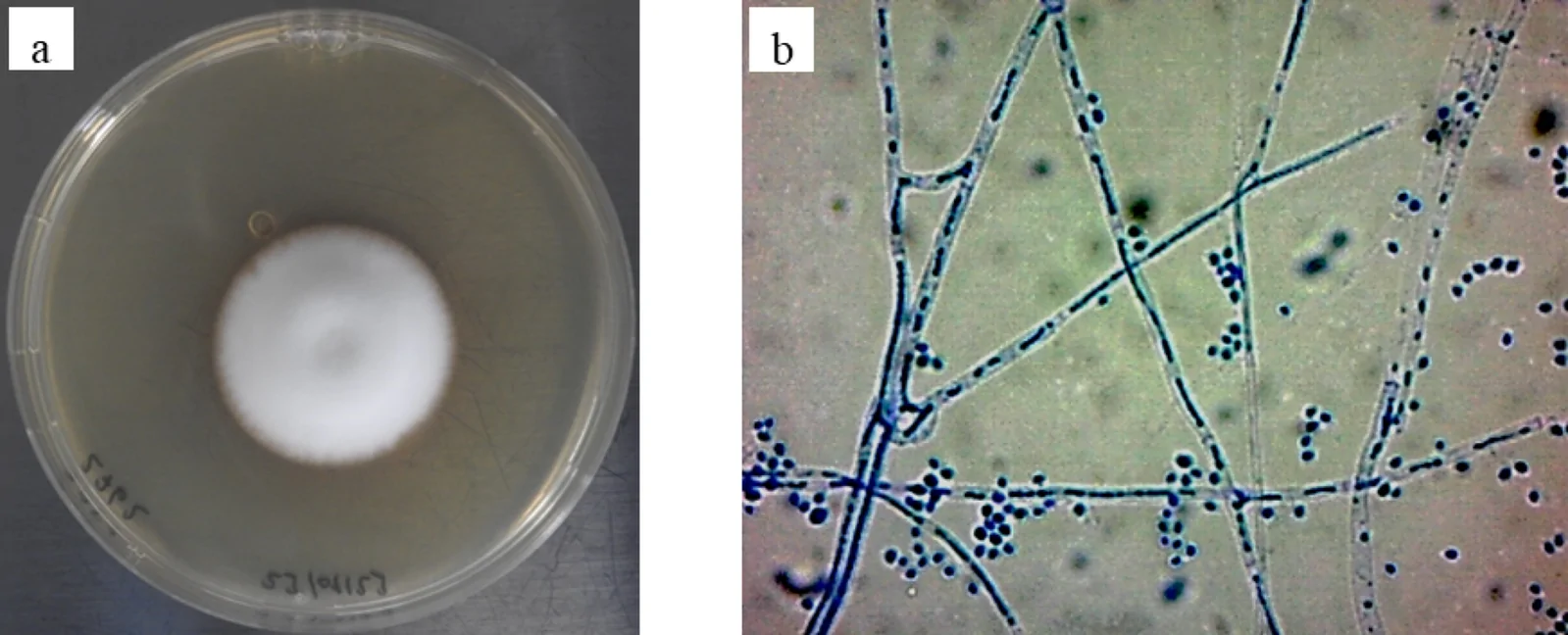
Colony morphology of (**a)** and conidia and hyphae of 27b2 (**b)**

**Fig. 3:**
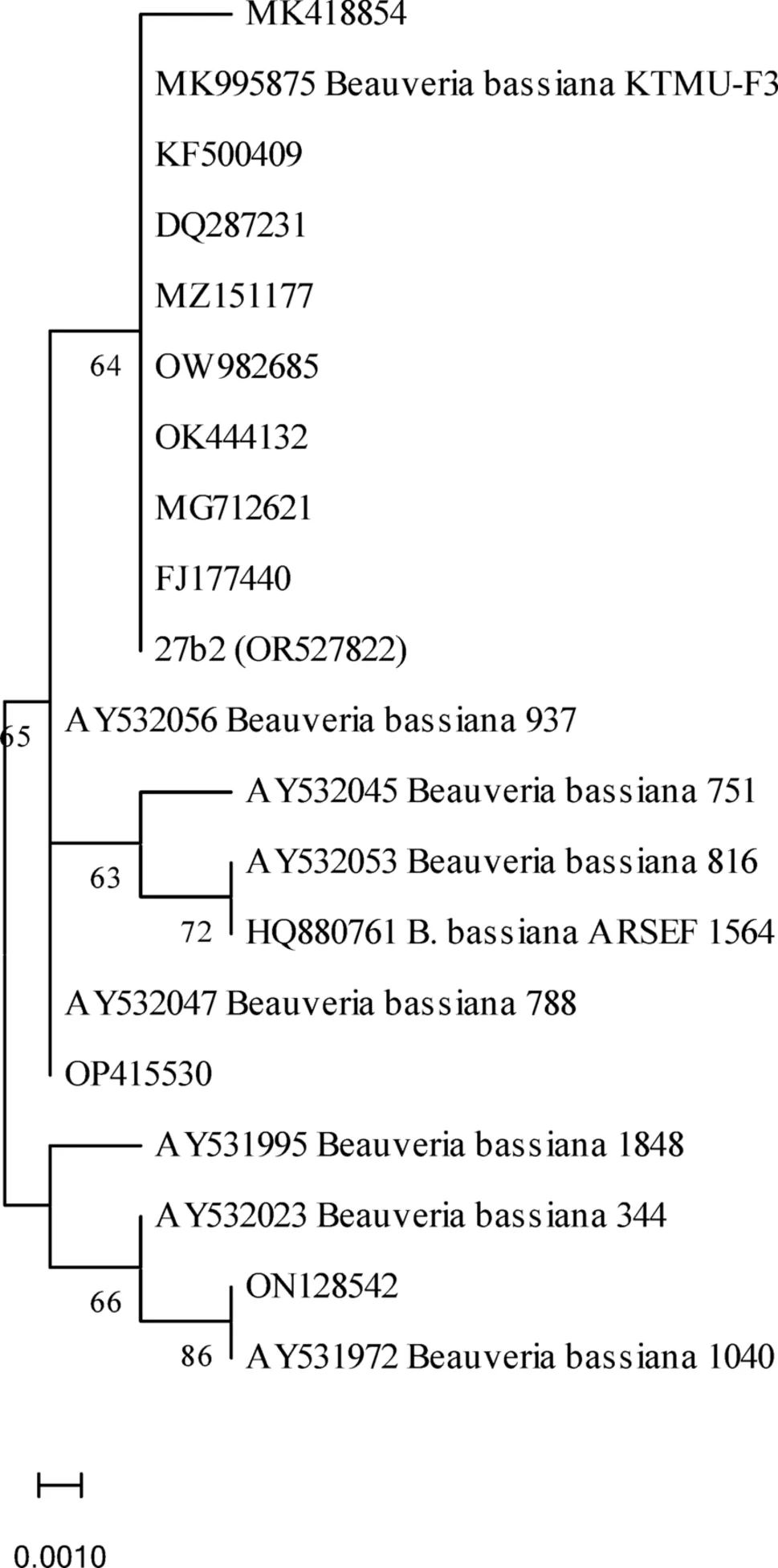
Phylogenetic tree based on fungal ITS was constructed by using the neighbor-joining method. The evolutionary distances were computed using the Kimura 2-parameter Method. The percentage of replicate trees in which the associated taxa clustered together in the bootstrap test (1000 replicates) are shown next to the branches

The fungi culture, which initially showed white, aerial mycelial growth on SDA medium, turned pale yellow after 7–10 days. In complex agar medium, the colonies of 27b2 are round and high-shaped and the surface has a powdery-cottony appearance (Fig. 2A). Microscopically, 27b2 has a transparent and branched hyphae structure and conidia varying from round to oval. The identification of fungal isolate 27b2 was confirmed by sequencing of the Internal Transcribed Spacer (ITS) rDNA. Results of phylogenetic analysis based on ITS1-5.8S rRNA-ITS2 showed that isolate 27b2 was closely related to *Beauveria bassiana* (Fig. 3). The nucleotide sequences (581 bp) were deposited in GenBank (OR527822).

### Insecticidal Effect of Fungal Extracts on *G. pyloalis* Larvae

**Fig. 4:**
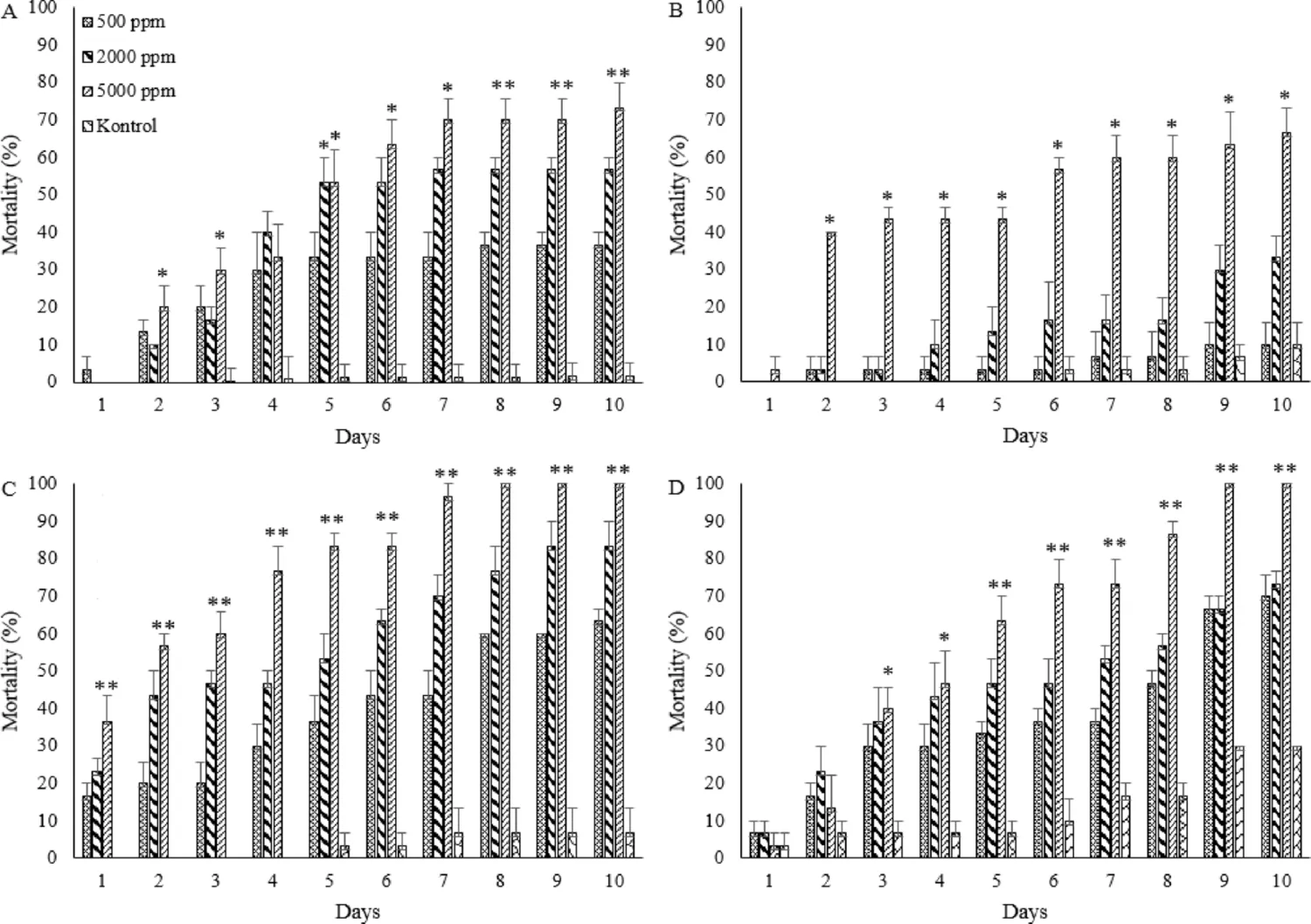
Mortality rates of different concentrations of the extracellular medium extract on insects (**A)** *G. pyloalis,* (**B)** *T. pityocampa*, (**C)** *D. micans* larvae, ** (D)** *D. micans* adult (*:(*p* < 0.05) and **:(*p* < 0.01))

**Fig. 5:**
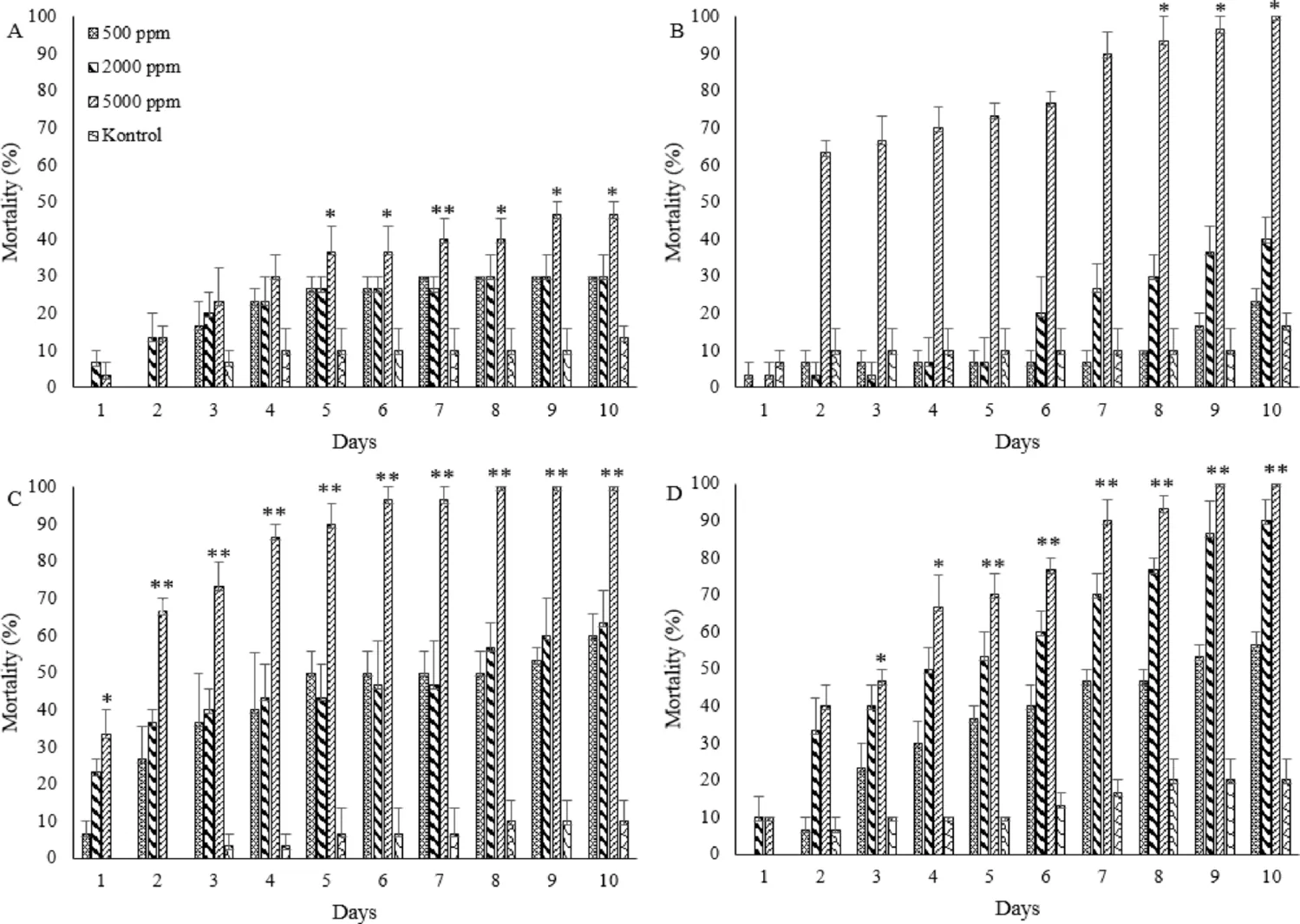
Mortality rates of different concentrations of mycelial extract on insects (**A)** *G. pyloalis,* (**B)** *T. pityocampa*, (**C)** *D. micans* larvae, (**D)** *D. micans* adult (*:(*p* < 0.05) and **:(*p* < 0.01))

The mortality rates of *G. pyloalis* larvae reached 30% on the 4th day and 37% on the 10th day at the 500 ppm concentration of the extracellular medium extract. At the other two concentrations (2000 and 5000 ppm), the mortality rates were 53% on the 5th day, 57% on the 10th day, and 73% on the 10th day (Fig. 4A), respectively. The mycelial extract exhibited a mortality rate of 17% and 30% at concentrations of 500 ppm after three and ten days, respectively. At concentrations of 2000 and 5000 ppm, mortality reached 30% after eight and five days, respectively. The highest mortality rate was observed on the 10th day of application at a concentration of 5000 ppm, reaching 47% (Fig. 5A). A statistically significant mortality rate (*p* < 0.05) was determined on the second and 5th days of application of both extracts at the highest concentration (5000 ppm) in comparison to the control. Specifically, for the extracellular medium extract, the analysis showed (χ^2^ = 0.99, df = 1, *p* < 0.05) and for the mycelial extract (χ^2^ = 0.64, df = 1, *p* < 0.05). The LC_50_ value of the effect of secondary metabolites obtained from the extracellular medium on *G. pyloalis* was calculated to be 2067 ppm. The LC_50_ value of the effect of metabolites obtained from micelles was calculated to be 31,087 ppm (Tables 1 and 2).

**Table 1:** The median lethal concentration (LC₅₀) of the insecticidal effects of the extracellular medium extract on the insects used in the study

	95% Cl						
Insects	LC_50_ (ppm)	Lower bound	Upper bound	Slope ± SE	LC_90_ (ppm)	df	X^2^
*D. micans* Larvae	394	146	1065	1,19 ± 0.22	4725	1	0.97
*D. micans* Adults	579	276	1213	1.67 ± 0.16	3858	1	0.7
*G. pyloalis* Larvae	2067	821	5204	1.16 ± 0.2	25,916	1	0.99
*T. pityocampa* Larvae	4211	2397	7395	2.25 ± 0.12	15,620	1	1

*Cl:* Confidence limits, *SE:* Standard error, *df:* degree of freedom, *X*^*2*^ : Chi-Square

**Table 2:** The median lethal concentration (LC₅₀) of the insecticidal effects of mycelium extract on the insects used in this study

	95% Cl						
Insects	LC_50_ (ppm)	Lower bound	Upper bound	Slope ± SE	LC_90_ (ppm)	df	X^2^
*D. micans* Larvae	717	264	1946	1.1 ± 0.22	11,212	1	0.7
*D. micans* Adults	559	295	1062	2.06 ± 0.14	2335	1	1
*G. pyloalis* Larvae	31,087	3676	262,843	0.52 ± 0.47	8,045,760	1	0.64
*T. pityocampa* Larvae	1730	1133	2642	3.21 ± 0.09	4654	1	0.84

*Cl:* Confidence limits, *SE:* Standard Error, *df:* degree of freedom, *X*^*2*^ : Chi-Square

### Insecticidal Effect of Fungal Extracts on *T. pityocampa* Larvae

The extracellular medium extract for *T. pityocampa* larvae did not result in mortality on the first day of the 500 and 2000 ppm applications. However, the mortality rate increased to 10% on the 9th day and the 4th day, respectively. In the case of the 5000 ppm application of extracellular medium extract, the mortality rate increased to 40% on the second day and continued to rise thereafter. On the final day of the experiment, the mortality rates were determined 10, 33, and 67%, respectively, in accordance with the increasing extract concentration (Fig. 4B). The mycelial extract applications at 500 and 2000 ppm resulted in mortality rates of 10 and 30% on the eighth day and 23 and 40% on the 10th day, respectively. At a concentration of 5000 ppm, the mortality rate reached 63% on the second day, 90% on the 7th day, and 100% on the 10th day (Fig. 5B). The mortality rates recorded on the 2nd and 8th days following the application of 5000 ppm extracts (extracellular media and mycelium) were higher compared to the control group (Extracellular: χ^2^ = 1, df = 1, *p* < 0.05; Mycelial: χ^2^ = 0.84, df = 1, *p* < 0.05). The LC_50_ value of the extracellular medium metabolites was determined 4211 ppm, while it was 1730 ppm in micellar medium metabolites (Tables 1 and 2).

### Insecticidal Effect of Fungal Extracts on *D. micans* Larvae and Adults

The research revealed the effectiveness of the extracellular medium extract from the *B. bassiana* (27b2) isolate in increasing the mortality rate of *D. micans* larvae. Moreover, the mortality rate was 43% on the 7th and second days, respectively, at doses of 500 and 2000 ppm. Also, the mortality rates significantly increased on the 10th day, reaching 63% and 83% respectively. At a concentration of 5000 ppm, the mortality rate was significantly increased to 57% on the second day, 97% on the 7th day, and 100% on the 10th day (Fig. 4C), respectively. The mycelial extract was applied at concentrations of 500 and 2000 ppm, resulting in a 40% mortality rate on the 4th and 3rd days, respectively, and a 60% mortality rate on the 10th and 9th days, respectively. Additionally, the mortality rate increased from 33% on the first day to 100% on the 8th day at a concentration of 5000 ppm (Fig. 5C). Notably, the mortality rates found for both extracts on the second day of usage at a dose of 5000 ppm were statistically different (*p* < 0.01) from those in the control group (Extracellular: χ^2^ = 0.97, df = 1; Mycelial: χ^2^ = 0.7, df = 1). The LC_50_ value of the effect of crude extracts taken from the extracellular medium on *D. micans* larvae was determined at 394 ppm, while the LC_50_ value of metabolites extracted from mycelium was 717 (Tables 1 and 2).

The mortality rates of fungal extracts on *D. micans* adults are demonstrated in Figs. 4D and 5D. The extracellular medium extract indicated a mortality rate of 47% on the 8th day at a concentration of 500 ppm and a 43% mortality rate on the 4th day at a concentration of 2000 ppm. In brief, 500 and 2000 ppm concentrations resulted in approximately 70% mortality on the 10th day. At a concentration of 5000 ppm, the mortality rate was 40% on the third day and reached 100% on the 9th day, as illustrated in Figs. 4D and 5D. The mycelial extract was applied at concentrations of 500, 2000, and 5000 ppm, resulting in mortality rates of 40% on the 6th day, 4th day, and second day, respectively. On the 10th day, the mortality rates were determined at 57% (500 ppm), 90% (2000 ppm), and 100% (5000 ppm). The highest concentrations of extracellular medium and mycelial extracts (5000 ppm) showed a statistically significant mortality effect on the 5th day of treatment, compared to the control group (Extracellular: χ^2^ = 0.7, df = 1, p < 0.01; Mycelial: χ^2^ = 1, df = 1, *p* < 0.01).An analysis of the LC_50_ values of extracts used to eliminate *D. micans* adults indicated that the extracellular medium had a value of 579 ppm, but the mycelium showed a value of 559 ppm (Tables 1 and 2).

## Discussion

The number of research examining the mortality effects of secondary metabolites extracted from *B. bassiana* observed against *G. pyloalis* is very limited. Lardeh and Zibaee [42] reported that beauvericin extracted from *B. bassiana* caused a decrease in nutrition in the 4th instar larvae of *G. pyloalis*. The larvae of *G. pyloalis* showed insufficient resistance to the metabolites obtained from isolate 27b2 to ensure the survival of the population. The metabolites potential for use in controlling this pest was significant despite the high LC_50_ value, as demonstrated by the statistical significance of the results.

A review of the literature revealed no studies investigating the lethal effect of secondary metabolites extracted from *B. bassiana* on *T. pityocampa*. This study represents the inaugural investigation of this nature. Sönmez et al. [43] indicated that 100% of larvae in the first, second, third, and fourth developmental stages died at increased application doses in their study of *B. bassiana* sporulation. Moreover, Özdemir et al. [44] conducted an experiment in which a spore suspension of *B. bassiana* was directly given to insects and the leaves that they consumed. The dose was 1 × 10^6^. At the end of the study, the LT_50_ value was determined to be 3.75 days in the insect application and 3.49 days in the leaf application. In the same application, the LT_90_ value was found 4.48 days in the insect application and 4.63 days in the leaf application [44]. The crude extracts used in this study produced the most effective outcomes on *T. pityocampa* following *D. micans*. In particular, it was observed that with the application of mycelium extract, insect larvae died completely within 10 days. The graphs were corroborated by the Probit analysis, which revealed that the LC values for *T. pityocampa* were significantly higher for the mycelial extracts than for the extracellular medium extracts. However, the metabolites obtained from both extractions also demonstrated appropriate results in terms of applicability.

The biological control of *D. micans*, one of the most prevalent harmful species in the Eastern Black Sea Region, has been the subject of ongoing research for several years [38, 39, 45]. A review of the literature revealed no studies investigating the insecticidal activity of secondary metabolites of *B. bassiana* on *D. micans*. In this regard, the originality of this study is further enhanced. In our study, the larvae of *D. micans* exhibited greater sensitivity than the adults to secondary metabolites extracted from the extracellular medium. Conversely, the facts differed in extracts derived from mycelium, revealing that *D. micans* adults showed more sensitivity than *D. micans* larvae to metabolites sourced from mycelium. However, the lethality rate of both extracts was higher in individuals of the *D. micans* species than in other species.

Nevertheless, a number of studies have been conducted with the objective of determining the effects of *B. bassiana* metabolites on different insect species. Daniel et al. [46] observed that methanolic and ethyl acetate-methanolic extracts of *B. bassiana* exhibited larvicidal activity against the third stage of *Aedes aegypti*. Also, Gurulingappa et al. found in their study that was conducted in 2011 that the methanolic mycelial extract and extracellular extract of *B. bassiana* had 80 and 97% lethal effects on *Aphis gossypii*, respectively [47].

Additionally, Abdullah [48] reported that the mortality rate of ethyl acetate-extracted *B. bassiana* extracellular medium on *Spodoptera littoralis* (Boisduval) was 86.7% at a concentration of 1000 ppm in 24 h and 100% on *A. gossypii.*

A study was conducted to ascertain the impact of crude soluble protein extracts derived from *B. bassiana* isolates on *S. littoralis* larvae. In this study, extracts were given to the alfalfa leaves that the larvae fed on and to the synthetic nutritional media of these larvae. This was achieved by applying the extracts to clover leaf discs or incorporating them into synthetic nutrient media. The results indicated that two isolates (01/12-Su and 01/88-Su) exhibited a mortality rate of 20–35% [49]. The impact of secondary metabolites derived from *B. bassiana* extracted with chloroform on cellular immune responses in *Eurygaster integriceps* (Puton) was also examined. The findings indicated that *B. bassiana* secondary metabolites significantly influenced the cellular immune response and PO activity [50].

In addition to crude extracts of *B. bassiana*, studies have also been conducted on the insecticidal effects of the identified metabolites of this fungus. Abdelgaleil et al. [51] isolated and purified a number of compounds from endophytic and soil fungal isolates, including phomaxanthone A and deacetylphomaxanthone (dimericxanthone) A, anthracobic acid (polyketide) A, and dehydroaustin (meroterpenoid). The study identified 6-epoxy-4-hydroxy-3-methoxy-5-methyl-cyclohexenone (cyclohexenone) metabolites and investigated their activity on larvae of *Culex pipiens* (Linnaeus) and *S. littoralis*. The findings indicated that the metabolites demonstrated both insecticidal and feeding-inhibitory activity on the larvae. Leckie et al. [52] determined that high mortality rates, along with delayed development and weight loss, were recorded in *Helicoverpa zea* (Boddie) larvae fed with foods containing *B. bassiana* mycelium. It was determined that the toxicity of mycelia or fungal metabolites can result in adverse effects, including mortality and delayed development, in insects. The *B. bassiana* 11–98 isolate was found to contain 3.75 mg of beauvericin per gram of dry mycelial weight [52].

GC–MS analysis of crude metabolites extracted from *B. bassiana* using ethyl acetate revealed six major chemical components: n-hexadecanoic acid (16.13%), 9,10-octadecadienoic acid (35.47%), 9-eicosyne (13.17%), n-heptacosane (8.36%), tetratetracontane (12.15%), and 7-hexyl eicosane (7.359%). These metabolites are reported to inhibit various esterase enzymes in insects, most notably acetylcholinesterase [53]. In another study, secondary metabolites such as oosporein, tenellin, and bassianin, extracted from *B. bassiana* mycelia using acetone, were found to exhibit cytotoxic effects on insects [54]. Overall, ethyl acetate and acetone are commonly used organic solvents for the extraction of secondary metabolites from B. bassiana in related studies.

Among the insect species tested, *D. micans* consistently exhibited higher susceptibility than the other species. In the experiment conducted with extracellular medium extracts, *D. micans* larvae showed the lowest LC_50_ value (394 ppm; χ^2^ = 0.97, df = 1), whereas in the assay using mycelium-derived metabolites, adults were found to be more susceptible than larvae (LC_50_ = 559 ppm; χ^2^ = 1, df = 1). This relative shift in susceptibility between developmental stages may be attributed to differences in cuticular permeability, detoxification enzyme activity, or physiological tolerance mechanisms, or differences in metabolite contents between extracellular medium extracts and mycelium-derived metabolites.

*G. pyloalis* larvae displayed the highest level of tolerance in both experiments, with particularly elevated LC_50_ and LC_90_ values recorded in the second assay (mycelium extract-containing) (LC_50_ = 31,087 ppm, LC_90_ = 8,045,760 ppm). The very wide confidence intervals and low slope values observed for this species indicate an extremely heterogeneous response within the population. Such heterogeneity may be associated with individual variability in susceptibility arising from genetic diversity, metabolic detoxification capacity, or behavioral differences. From an applied perspective, the high concentrations required to achieve 90% mortality suggest that the tested fungus may have limited effectiveness against *G. pyloalis* larvae under field conditions.

Larvae of *T. pityocampa* exhibited a moderate level of susceptibility in both experiments. The relatively high slope values (up to 3.21 ± 0.09 for mycelium extract) indicate that small increases in dose resulted in rapid increases in mortality, a characteristic generally considered desirable for pest management applications. In addition, the narrower confidence intervals calculated for *T. pityocampa* support the reliability of the estimated lethal concentration values.

Overall, the low chi-square (χ^2^) values obtained across all test groups confirm a good fit of the probit model to the observed data and indicate that the dose–response analyses are robust and reliable. These statistical parameters, when compared to control groups (*p* < 0.05 or *p* < 0.01), validate the insecticidal potential of the extracts. Taken together, the results suggest that the tested fungus shows promising potential for the control of *D. micans* and, to a lesser extent, *T. pityocampa*, whereas its efficacy against *G. pyloalis* larvae appears to be limited.

## Conclusion

The research presents conclusive evidence of the harmful effect of crude extracts from *B. bassiana* 27b2, derived from forest pests (*D. micans* and *I. sexdentatus*) utilizing two distinct organic solvents, against all three insect species examined. The extracellular medium extract obtained using ethyl acetate and the mycelial extract obtained using acetone were found to be lethal to both larvae and adults of *D. micans*, with 100% susceptibility. Furthermore, the acetone extract demonstrated 100% lethal activity on *T. pityocampa* larvae, indicating its potential as a bio-pesticide. The utilization of secondary metabolites derived from entomopathogenic organisms represents a viable alternative to the direct use of the organism itself as a means of insect control. This is particularly the case in unfavorable conditions, such as high temperatures, drought, and the presence of chemical pesticides. The results of the present study present significant indications that secondary metabolites from *B. bassiana*, an entomopathogenic fungus with the ability to infect around 1.000 insect species, will be deadly to other detrimental insects.

These extracted secondary metabolites can be sprayed on plants that are infested by pests to ensure that they are eaten by pests. By doing so, the negative effects of these metabolites on beneficial insects that are predators or parasitoids of the pest are also prevented. In other words, this method is considered to be more effective than the direct application of the fungus spores in preventing the beneficial insects from being harmed. It is anticipated that the results of the present study will be inspiring for future studies and will be the basis for future field studies.

In future studies, conducting compositional analyses of the extracted metabolites and evaluating their applications under controlled conditions in natural habitats or directly in field environments will enhance the effectiveness and scientific value of such research.

## Data Availability

All the data/information can be obtained from the first author by email, if necessary.
